# PhenoFly Planning Tool: flight planning for high-resolution optical remote sensing with unmanned areal systems

**DOI:** 10.1186/s13007-018-0376-6

**Published:** 2018-12-21

**Authors:** Lukas Roth, Andreas Hund, Helge Aasen

**Affiliations:** Institute of Agricultural Sciences, ETH Zurich, Universitätstrasse 2, 8092 Zurich, Switzerland

**Keywords:** High-throughput phenotyping, Flight planning, Low-altitude remote sensing, Mapping from imagery, Viewing geometry, Ground control point (GCP)

## Abstract

**Background:**

Driven by a huge improvement in automation, unmanned areal systems (UAS) are increasingly used for field observations and high-throughput phenotyping. Today, the bottleneck does not lie in the ability to fly a drone anymore, but rather in the appropriate flight planning to capture images with sufficient quality. Proper flight preparation for photography with digital frame cameras should include relevant concepts such as view, sharpness and exposure calculations. Additionally, if mapping areas with UASs, one has to consider concepts related to ground control points (GCPs), viewing geometry and way-point flights. Unfortunately, non of the available flight planning tools covers all these aspects.

**Results:**

We give an overview of concepts related to flight preparation, present the newly developed open source software *PhenoFly Planning Tool*, and evaluate other recent flight planning tools. We find that current flight planning and mapping tools strongly focus on vendor-specific solutions and mostly ignore basic photographic properties—our comparison shows, for example, that only two out of thirteen evaluated tools consider motion blur restrictions, and none of them depth of field limits. In contrast, *PhenoFly Planning Tool* enhances recent sophisticated UAS and autopilot systems with an optical remote sensing workflow that respects photographic concepts. The tool can assist in selecting the right equipment for your needs, experimenting with different flight settings to test the performance of the resulting imagery, preparing the field and GCP setup, and generating a flight path that can be exported as waypoints to be uploaded to an UAS.

**Conclusion:**

By considering the introduced concepts, uncertainty in UAS-based remote sensing and high-throughput phenotyping may be considerably reduced. The presented software *PhenoFly Planning Tool* (https://shiny.usys.ethz.ch/PhenoFlyPlanningTool) helps users to comprehend and apply these concepts.

## Background

Unmanned areal systems (UAS) are increasingly used as platforms to monitor vegetation by means of optical remote sensing [1, 2]. Recent UAS and sensor technologies allow ground sampling distances (GSD) in the centimeter [3], millimeter [4] or even sub-millimeter range [5]. This development enables identification of details on the plant or even plant organ level. Especially in crop monitoring, the trend for finer resolved photos has led to an increasing number of applications [6]. GSDs $\le$ 0.1 m were sufficient for empirical estimations of general crop characteristics such as leaf area index [3, 7, 8], crop nitrogen content [3, 7–9], and ripening processes [10], or weed detection [11, 12]. GSDs $\le$ 0.02 m served as base for structure-from-motion (SfM) processing followed by plant height extraction [13–18]; a method that was used to subsequently estimate plant biomass, lodging, yield, and other plant height related parameters. The segmentation of images in pixels related to either plants or soil demands comparable GSDs $\le$ 0.01 m and allowed to measure canopy cover, leaf area index [19] and crop density [4, 19, 20]. On the very extreme, GSDs ${<}\,$ 0.001 m paved the way for recent works that estimated seed emergence [5, 21].

Nevertheless, beside this remarkable achievements, reports about failed remote sensing missions with UASs are frequent. It is not enough just to buy a high-resolution camera, set it to automatic mode and fly at low altitude: proper flight preparation is essential [22] and refraining from doing so is the main source of failure in UAS based remote sensing [23]. Based on our own experience and a literature review, we spotted two main error sources: inadequate settings of camera parameters, and sub-optimal settings of the mapping mission. Even in successful studies, crucial parameters were frequently not reported and might not have been considered. For example, flight speed and camera settings are essential to prevent motion blur if performing high-resolution optical remote sensing. Nevertheless, only three of the mentioned studies provided values for flight speed [7, 19, 21], and only four provided camera parameters including shutter speed and aperture settings [3, 15, 16, 19]. Regrettably, our own publications form no exception [e.g. missing flight speed and/or shutter speed in 7, 16, 18]. Presumably the most common cause for this sub-optimal performance is the tendency of UAS pilots to set cameras to automatic settings “to minimize experiment complexity” [20].

Proper camera and flight mission settings are in our opinion essential to reduce uncertainty in optical remote sensing with UASs. With this publication, we like to provide a tool to master the aforementioned complexity. We thereby assume that the difficulty arises mainly from the decoupling between UAS, camera system, and processing software. A very comprehensible introduction in camera settings for areal surveys was given by [24]. For the available flight planning software on the other hand, most tools are specifically designed for certain all-in-one UAS solutions or for photogrammetric products. Nevertheless, UAS and camera systems for vegetation monitoring are rarely out-of-the-box solutions, and high costs may still talk in favor for custom-build alternatives. In addition, we believe that the requirements—based on the targeted objects to measure—should drive the purchase, not otherwise.

The scope of this publication therefore includes major relevant concepts and parameters in optical remote sensing and mapping with UASs, and we start with a brief overview of them. We then link and integrate the mentioned concepts in a vendor- and software package independent flight planning tool called *PhenoFly Planning Tool*. We furthermore contrast the presented software with other comparable tools. In a last step, we demonstrate a practical implementation of a close-range mapping flight using *PhenoFly Planning Tool* in combination with recent UAS and photography technology.

### Photography with digital frame cameras

The main component in photography is the imaging device. It may capture a two-dimensional image (digital frame camera), a one-dimensional image (pushbroom scanner) or a point image (flying spot scanner) [22]. In this publication and the presented software *PhenoFly Planning Tool*, we focus on digital frame cameras equipped with a lens system. In the following, only the most relevant parameters are briefly introduced (Table 1). Further physical and technical background can be found in the corresponding literature [e.g. 25].

**Table 1:** List of terms and corresponding symbols used in photography with digital frame cameras and related input and output categories in the software *PhenoFly Planning Tool*

Term	Symbol	$\rightarrow$ Input/$\leftarrow$ output
Sensor		
Sensor size (x/y-axis)	$S_{x'}, \; S_{{y'}}$	$\rightarrow$ Sensor/lens
Number of recorded pixels (x/y-axis)	$S_{{a}}, \; S_{{b}}$	$\rightarrow$ Sensor/lens
Distance between pixel centers	$S_{\delta }$	$\leftarrow$ Sensor/lens
Circle of confusion limit	*c*	$\leftarrow$ Sensor/lens
Lens		
Focal length	*f*	$\rightarrow$ Sensor/lens
Aperture (f-number)	*N*	$\rightarrow$ Sensor/lens
Angle of view (x/y-axis)	${\text {AOV}}_x, \; {\text {AOV}}_y$	$\leftarrow$ Photography
Hyperfocal distance	*H*	$\leftarrow$ Photography
Diffraction limit	*d*	$\leftarrow$ Photography
View		
Flight height	*h*	$\leftrightarrow$ Imaging
Ground field of view (x/y-axis)	$G_x, \; G_y$	$\leftarrow$ Photography
Ground sampling distance	${\text {GSD}}$	$\leftrightarrow$ Imaging
Sharpness		
Focus distance	*s*	$\leftarrow$ Photography
Depth of field (near/far)	$D_{{\text {N}}}, \; D_{{\text {F}}}$	$\leftarrow$ Photography
Exposure		
Exposure value	EV	$\rightarrow$ Imaging
Shutter speed	$I_{t}$	$\rightarrow$ Imaging
Film speed	ISO	$\leftarrow$ Imaging
Maximum photo trigger frequency	${I_{f, \max }}$	$\rightarrow$ Sensor/lens

#### Sensor

**Fig. 1:**
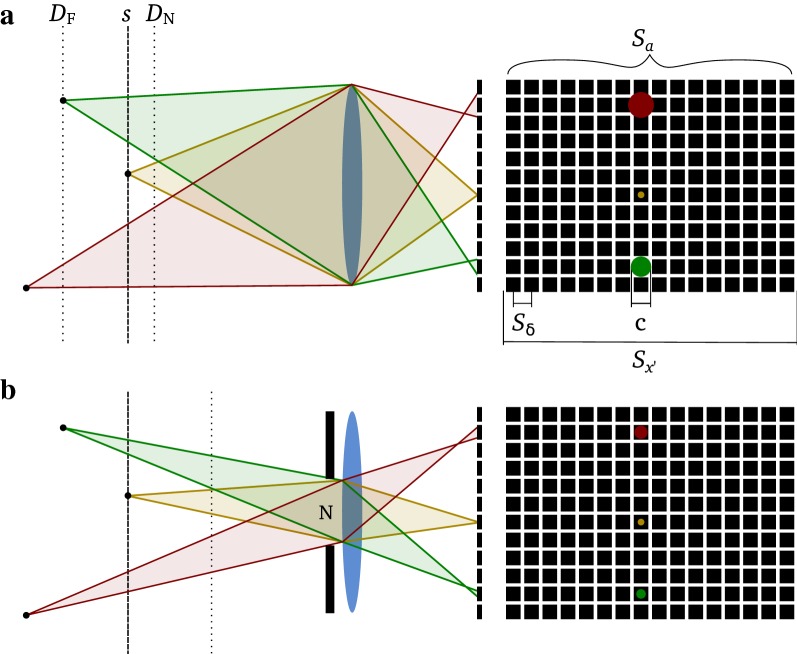
Concepts in digital frame camera photography: **a** Thin lens model and corresponding depth of field with focus distance (*s*), near and far depth of field limits ($D_{{\text {N}}}$/$D_{{\text {F}}}$) and imaged points (red, brown and green) on schematic drawing of sensor with size ($S_{x'}$) and number of recorded pixels ($S_a$) on x-axis, distance between pixel centers ($S_\delta$) and circle of confusion limit (*c*). **b** Same lens as in **a** but with closed aperture (*N*)

According to [25], major intrinsic characteristics of a digital frame camera are determined by the sensor, namely by its size ($S_{x'} \times S_{y'}$) and number of recorded pixels ($S_a \times S_b$) (Fig. 1a). The distance between pixel centers ($S_{\delta }$) is defined as the ratio between sensor size and number of recorded pixels. In the following, we assume a common pixel aspect ratio of one [25]. Therefore, $S_\delta$ is defined as

1 $$\begin{aligned} S_\delta = \frac{S_{x'}}{S_a} = \frac{S_{y'}}{S_b}. \end{aligned}$$

Ideally, large distances between pixel centers correspond with larger physical pixels and therefore increase photon gain, providing a higher signal-to-noise ratio and dynamic range. Smaller distances between pixel centers on the other hand allow higher numbers of recorded pixels per sensor axis and therefore increase the total number of recorded pixels [25]. One may therefore tend to combine large pixel sizes with large sensor sizes to optimize both number of recorded pixels and signal-to-noise ratio and dynamic range properties. Nevertheless, larger sensor sizes require bulkier lens systems and therefore contradict the need for small payloads for UASs.

For an imaging system in remote sensing, sharpness is crucial. In photography, the concept of the circle of confusion is used to describe an acceptably sharp image (Fig. 1a, b). The circle of confusion is the spot that point source rays form on an image when the lens is not perfectly in focus. While in artistic photography, the circle of confusion limit (*c*) is defined as the largest blur spot that a human eye perceives as a single point in the final product [26], for image processing it is defined as the distance between pixel centers, the ultimate limit of the sensing system. It is therefore proposed that the circle of confusion limit should be smaller than or equal to the distance between pixel centers [27],

2 $$\begin{aligned} c \le S_{{\delta }}. \end{aligned}$$

An additional parameter to consider is the image file format. RAW file formats offer full functionality to preserve the quality of the signal captured by a sensor, but have the drawback of a lack of standardization [25]. Standardized, lossless compressed formats such as TIFF, Lossless JPEG or PNG on the other hand ensure interchangeability and accessibility of data, but may reduce the information content of remote sensing images, for example by reducing color depth to 8 bit, demosaicing a Bayer-type sensor image, or performing irreversible color balance adjustments (often called white balance) [25]. Formats using lossy compression such as JPEG may not represent a valid option for remote sensing applications, as the level of image degradation increases with increasing compression [25].

#### Lens

For most common frame cameras, a lens compound complements the sensor to an imaging device. Lens compounds may be categorized in lenses where the thickness is negligible (thin lenses) and those where the thickness is not negligible (thick lenses) [22]. In the following, for simplicity we focus on thin lenses only. A thin lens is characterized by its focal length (*f*) and aperture f-number (*N*) [25]. Focal length and aperture may be immutable or mutable, depending on the lens type. Often lens specifications refer to a “35 mm equivalent focal length” or a “crop factor”, terms that relate lens characteristics to analogue 35 mm film cameras [24]. In the following, we use the term focal length to refer to the effective, physical focal length.

The lens aperture f-number and focal length in combination with the sensor result in an additional characteristic of the lens—the hyperfocal distance (*H*). The hyperfocal distance is defined as the focus distance beyond which all imaged objects are not restricted by the circle of confusion limit and therefore regarded as sharp [25],

3 $$\begin{aligned} H = \frac{f^2}{N \cdot c} + f. \end{aligned}$$

The hyperfocal distance varies as a function of the lens aperture. The other two parameters (sensor-specific circle of confusion limit and the focal length of the lens) are fixed constants of the camera system.

Another function of the lens aperture represents the diffraction limit: for very small apertures, the small opening may deflect incoming rays and therefore reduce sharpness due to light diffraction. The diffraction limit (*d*) is calculated as

4 $$\begin{aligned} d = 2 \cdot 1.22 \lambda \cdot N , \end{aligned}$$

where $\lambda$ is the wavelength of light [24]. The imaging device is diffraction limited if *N* is chosen in a way that the diffraction limit is bigger that the circle of confusion limit, $d > c$.

#### View

The imaging geometry of a digital frame camera strongly depends on the orientation of the camera. In the following, we assume that the camera is oriented in a nadir view and that the terrain is perfectly planar, a common simplification in remote sensing [22].

**Fig. 2:**
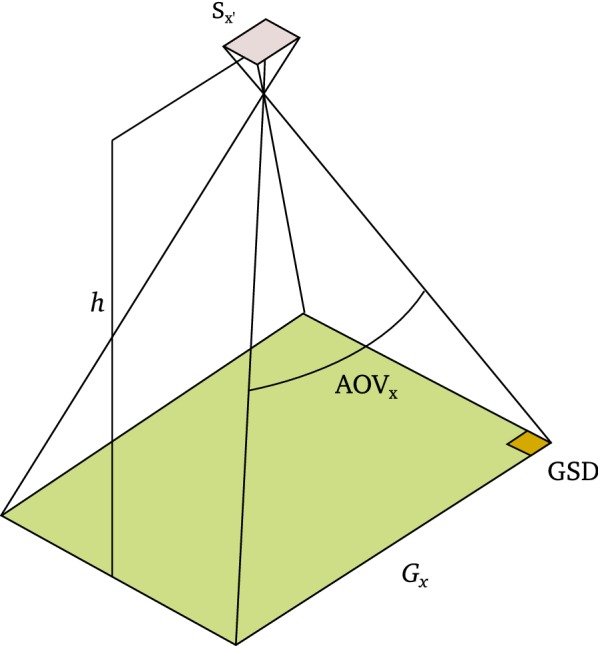
Concepts in digital frame camera photography: angle of view (AOV), ground field of view (*G*) and ground sampling distance covered by a sensor pixel (GSD) depending on flight height (*h*) and sensor size (*S*)

If combining the focal length with the sensor size, one can derive the angle of view (AOV) (Fig. 2) [25] as

5 $$\begin{aligned} {\text {AOV}}_x = 2 \cdot \tan ^{-1}\left( \frac{S_{x'}}{2 \cdot f}\right) , \quad {\text {AOV}}_y = 2 \cdot \tan ^{-1}\left( \frac{S_{y'}}{2 \cdot f}\right) . \end{aligned}$$

In nadir orientation, the view of a frame camera is determined by the distance to the object, which corresponds to the flight height (*h*) over ground. A certain flight height results in a specific ground field of view (*G*) (sometimes also called spatial support), the area that is visible in one photo,

6 $$\begin{aligned} G_x = S_{x'} \cdot \frac{h}{f} , \quad G_y = S_{y'} \cdot \frac{h}{f}. \end{aligned}$$

The corresponding ground sampling distance (GSD) if assuming a pixel aspect ratio equal one is defined as

7 $$\begin{aligned} {\text {GSD}} = \frac{G_x}{S_a} = \frac{G_y}{S_b}, \end{aligned}$$

Leachtenauer and Driggers [28] and represents the area on the ground covered by one pixel. GSD is an indicator for the minimum size of a detail that is still resolved in an image, and low GSDs may prevent successful feature extraction in images. Torralba [29] demonstrated that humans can recognize up to five objects with 80% accuracy in thumbnails of only 32 $\times$ 32 pixel. Based on this finding, [24] recommend that the features to extract should have a sizes of at least 5 $\times$ GSD. We experienced that for segmentation in plant and soil, leaf sizes of 3 $\times$ GSD may already be sufficient [19].

#### Sharpness

Imaged objects are only regarded as sharp if the circle of confusion limit is respected (see section ‘Sensor’). For a specific focus distance, one can calculate the depth of field, characterized by the minimum and maximum distance of objects that are still regarded as sharply imaged (Fig. 1a). The depth of field is calculated as

8 $$\begin{aligned} D_{{\text {N,F}}}={\frac{s\cdot H}{H\pm s}}, \end{aligned}$$

where *s* is the focus distance, $D_{{\text {N}}}$ is the near limit and $D_{{\text {F}}}$ the far limit of the depth of field [30]. For UAS based photography, it is favorable to have a depth of field that expands equally before and beyond the distance to the ground and spans the vertical extend of objects of interest,

9 $$\begin{aligned} h = \frac{D_{{\text {N}}} + D_{{\text {F}}}}{2}. \end{aligned}$$

Therefore, if combining Eqs. 8 and 9, one can calculate the optimal focus distance as

10 $$\begin{aligned} s = \frac{H \sqrt{h}}{\sqrt{H + h}}. \end{aligned}$$

#### Exposure

The exposure of a frame camera is controlled by the shutter speed ($I_t$), the aperture f-number (*N*) and sensor sensitivity, for example expressed as film speed ISO [25]. In the following, we focus on main concepts related to correct exposure. For a more thorough discussion of exposure and its influence on signal-to-noise ratio, dynamic range, depth of field, motion blur, and diffraction, we refer to [24].

A common way to summarize and compare exposure configurations is to use exposure values (EV) [26], defined as

11 $$\begin{aligned} {\text {EV}} = \log _{2}{\frac{N^{2}}{I_t}} + \log _{2}{{\frac{100}{{\text {ISO}}}}}. \end{aligned}$$

The aperture setting strongly influences the depth of view (see section ‘Sharpness’), but also the resolving power of the lens [24, 26]. Therefore, the aperture should be set independently from the lighting situation in a way that the setting is close to the optimal aperture opening (specific for each lens), but also provides an adequately sized depth of field suitable for the remote sensing purpose.

The shutter speed on the other side is in close relation to the flight speed: we experienced that wrong shutter speed settings are a major cause of motion blur. Therefore, to prevent motion blur, the shutter speed should be in alignment with the flight speed (see following section ‘Mapping areas’). Nevertheless, if increasing the shutter speed one may also need to open the aperture to compensate for the shorter integration time.

As a consequence, the remaining adjustable parameter in Eq. 11 is the sensor sensitivity ISO. If measuring the exposure value needed for a certain situation, one can derive the optimal ISO setting with

12 $$\begin{aligned} {\text {ISO}} = \frac{25 N^2 \cdot 2^{2-{\text {EV}}}}{I_t}. \end{aligned}$$

Nevertheless, one should consider the maximal tolerable signal-to-noise ratio and therefore maximum tolerable ISO setting of the sensor. Recommendation from benchmark tests are often publicly available [24, e.g. https://www.dxomark.com]. If the maximum tolerable ISO value is exceeded, one has to reduce shutter speed and, to prevent motion blur, flight speed, or increase the aperture opening.

A common practice is to validate exposure settings before the mapping flight by positioning the UAS over a representative scene (e.g. the center of a field experiment) and display the intensity histogram of the camera. If no accumulation of counts in border bins is visible while the intensity peaks are located at center bins, one may regard the exposure as optimized. This practice assumes that the dynamic range of the sensor is large enough to cover the extremes of the scene (dark regions as well as bright spots), so that no information is lost due to under- or overexposure.

### Mapping areas with unmanned areal systems

Mapping areas using aerial photography is a well established task in photogrammetry. For further details on the topic please visit the corresponding literature [e.g. [22]]. In the following, we focus on parameters that are specific for UAS photography (Table 2).

**Table 2:** List of terms and corresponding symbols used in mapping areas with unmanned areal systems (UASs) and related input and output categories in the software *PhenoFly Planning Tool*

Term	Symbol	$\rightarrow$ Input/$\leftarrow$ output
Mapping areas		
Mapping area (width/depth)	$A_x, A_y$	$\rightarrow$ Mapping
Ground field of view (along/across flight dir.)	$G_{{E}}, G_{S}$	$\leftarrow$ Mapping
Spacing between exposures	*E*	$\leftrightarrow$ Mapping
Spacing between flight lines	*S*	$\leftrightarrow$ Mapping
Percent end lap	$E_\%$	$\leftrightarrow$ Mapping
Percent side lap	$S_\%$	$\leftrightarrow$ Mapping
Number of exposures per flight line	${E}_n$	$\leftarrow$ Mapping
Number of flight lines	${S}_n$	$\leftarrow$ Mapping
Photo trigger frequency	$I_{f}$	$\leftarrow$ Mapping
Number of photos	$I_n$	$\leftarrow$ Mapping
Exposure position of photos	$I_{{\mathrm {Pos}}}$	$\leftarrow$ Mapping
Flight speed	$F_v$	$\leftarrow$ Mapping
Flight duration	$F_t$	$\leftarrow$ Mapping
Motion blur	$\delta$	$\rightarrow$ Mapping
Plot center position (x/y-axis)	$p_{{\mathrm {Pos}},x}, p_{{\mathrm {Pos}},y}$	$\leftarrow$ Mapping
Ground control points (GCPs)		
Number of GCPs (x/y-axis)	${\text {GCP}}_{n,x}, {\text {GCP}}_{n,y}$	$\rightarrow$ GCPs
Position of GCPs (x/y-axis)	${\text {GCP}}_{{\mathrm {Pos}},x}, {\text {GCP}}_{{\mathrm {Pos}},y}$	$\leftarrow$ Mapping
GCP arrangement pattern		$\rightarrow$ GCPs
GCP recover frequency in photos	$\nu _{{\text {GCP}}}(k)$	$\leftrightarrow$ Mapping
Viewing geometry		
Positioning precision (standard deviation)	$\sigma _{I_{{\mathrm {Pos}}}}$	$\rightarrow$ Mapping
Zenith angle frequency	$\nu _\theta (\theta )$	$\leftarrow$ Viewing geometry
Way-point flight mapping missions		
Maximum flight duration	$F_{t, \max }$	$\rightarrow$ Sensor/lens
Start location (Lat, Long)	${P}_{{\mathrm {Start}}}$	$\rightarrow$ Location
Mapping area edge location (Lat, Long)	${P}_{{\mathrm {Edge1}}}$	$\rightarrow$ Location
Flight direction location (lat, long)	${P}_{{\mathrm {Dir}}}$	$\rightarrow$ Location
Maximum number of way-points	$P_{n,\max }$	$\rightarrow$ Location

#### Mapping areas

For simplicity, we restrict this publication and the software *PhenoFly Planning Tool* to squared mapping areas. A squared mapping area is defined by its width and depth ($A_x \times A_y$). In UAS based photography, this area is typically substantially larger than the ground field of view (*G*) of the imaging device. As a consequence, the mapping flight includes sequential exposure stations and results in multiple photos per mapping area. Photogrammetry techniques allow to align adjacent photos to determine the exact exposure position, and to produce a digital mosaic [22]. Basic perquisites for these techniques are overlaps between photos, expressed as overlap between flight lines (percent side lap, ${S_\%}$) and overlap in flight direction (percent end lap, ${E_\%}$). These overlaps can be transformed in spacing between exposures (*E*) and spacing between flight lines (*S*),

13 $$\begin{aligned} E&= G_E \left( 1- \frac{{E_\%}}{100} \right) , \end{aligned}$$

14 $$\begin{aligned} S&= G_S \left( 1 - \frac{{S_\%}}{100} \right) , \end{aligned}$$

where $G_E$ is the ground field of view in flight direction and $G_S$ across flight direction. Depending on the orientation of the camera, $G_E$ and $G_S$ are defined as

15 $$\begin{aligned} G_E&= G_y, \end{aligned}$$

16 $$\begin{aligned} G_S&= G_x, \end{aligned}$$

for $S_{y'}$ in flight direction and

17 $$\begin{aligned} G_E&= G_x, \end{aligned}$$

18 $$\begin{aligned} G_S&= G_y, \end{aligned}$$

for $S_{x'}$ in flight direction. The requirement for overlaps depends on the intended use—while in classical areal photography, end laps between 55 and 65% and side laps of 30% are recommended [22], SfM package manufacturer usually recommend values beyond 85% for end laps and 70% for side laps [e.g. 31].

Overlap in the SfM process is needed for feature-based and dense matching of images [32]. Feature-based matching allows a bundle adjustment of images to determine exposure positions and optionally intrinsic camera parameters [33]. Dense matching allows the generation of a dense point cloud based on exposure positions, intrinsic camera parameters and image content [34].

**Fig. 3:**
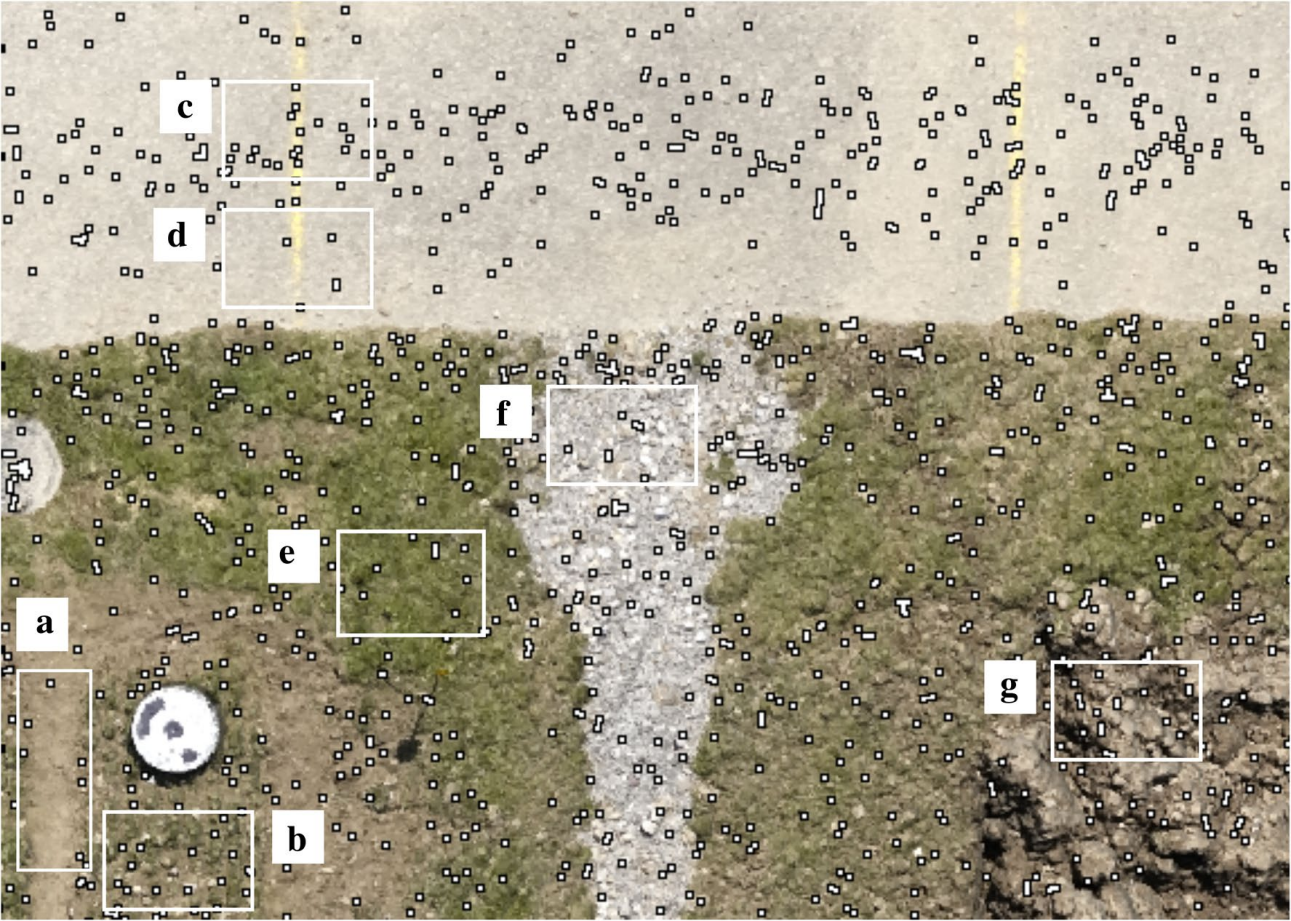
Detected tie points for a section of Experiment 3. **a** Driving lane of field experiment. **b** Experimental wheat plot. **c**, **d** Street. **e** Meadow. **f** Drainage. **g** Tilled soil

For the bundle adjustment, a theoretical minimum of three matching features between adjacent images (tie points) is needed [22]. Although small, this requirement may already represent a limit if mapping very homogeneous fields with few detectable and reliable features, for example bare soil with weak texture or artificial surfaces (Fig. 3a, d). Nevertheless, according to our own experience, variation in ground coverage in field experiments offer sufficient detectable features (Fig. 3b, c, e–g) if using recommended overlaps of SfM package manufacturer. Still, poor image quality may influence the success of feature-based image matching.

For the dense point cloud generation, images with a high overlap allow very dense resulting point clouds [32]. Nevertheless, [34] showed that complex vegetation as well as surfaces with homogeneous texture lead to sparser areas in dense clouds, calling for even higher overlaps than recommended by SfM package manufacturer.

In addition to overlap values, one should also ensure that the required overlaps cover the whole mapping area, including the extremes. As a consequence, one needs to extend the mapping area in both dimensions and directions by half of the corresponding ground field of view. The resulting number of exposures per flight line (${E_n}$) and number of flight lines (${S_n}$) is calculated as

19 $$\begin{aligned} E_n&= {\text {ceil}} \left( \frac{A_y + G_E}{E} \right) , \end{aligned}$$

20 $$\begin{aligned} S_n&= {\text {ceil}} \left( \frac{A_x + G_S}{S} \right) , \end{aligned}$$

where ${\text {ceil}}(x)$ is a ceiling function that maps *x* to the least integer greater than or equal to *x*. The number of photos per mapping area is therefore defined as

21 $$\begin{aligned} I_n = E_n \cdot S_n, \end{aligned}$$

and should not exceed the maximum storage capacity of the imaging device.

When the number and spacing of flight lines is known, one can approximate the minimum flight speed ($F_{v,\min }$) required to complete the flight in the maximum allowed flight duration ($F_{t,\max }$),

22 $$\begin{aligned} F_{v,\min } = \frac{S_n \cdot (A_y + G_S ) + A_x + G_E}{F_{t,\max }} . \end{aligned}$$

While the flight duration determines the minimum flight speed $(F_{v,\min })$, the maximum flight speed( $F_{v,\max }$) is determined by the fastest photo triggering frequency ($I_{f, \max }$) that the optical system supports,

23 $$\begin{aligned} F_{v,\max } = I_{f, \max } \cdot E. \end{aligned}$$

The chosen flight speed ($F_v$) should therefore range between the two mentioned limits,

24 $$\begin{aligned}&F_{v,\min } \le F_{v} \le F_{v,\max }. \end{aligned}$$

Another factor to consider is motion blur ($\delta$), usually denoted in percentage of the size of a pixel and caused by moving objects during one exposure [25]. A long shutter time in combination with a fast flight speed may force motion blur. O’Connor et al. [24] proposed to keep motion blur ${<}\,150\%$. Nevertheless, based on positive experiences with motion blur values ${<}\,50\%$ for plant and soil segmentation in [19] and motion blur values ${<}\,10\%$ for automatic GCP detection in present, unpublished own research, we recommend to keep motion blur as low as possible, but at least ${<}\,50\%$. The flight speed should then be chosen based on the maximum tolerable motion blur,

25 $$\begin{aligned} F_{v} = \frac{{\text {GSD}} \cdot \delta }{ I_t}, \end{aligned}$$

while also considering the limits based on Eq. 24.

#### Ground control points

Ground control points (GCPs) are used to gain information about exposure orientation and position of photos [22]. GCPs are an implied standard to process digital frame camera photos made with UASs [18, 35–39]. The number (${\text {GCP}}_{n}$) and placement pattern (${\text {GCP}}_{{\mathrm {Pos}}}$) of GCPs thereby determines the frequency of GCP recoveries in photos ($\nu _{{\text {GCP}}}$).

Depending on the purpose of the mapping campaign, the requirements on recover frequencies may differ: [35] demonstrated that one visible GCP per image is sufficient for accurate georeferencing if performing aero-triangulation with manual tie points. Mesas-Carrascosa et al. [40] and Gerke and Przybilla [38] could show that for SfM products, the distribution pattern and spacing of GCPs is of major importance too. Harwin et al. [37] noticed a stronger degradation of the vertical precision than of the horizontal precision of SfM products if reducing GCPs from approximately one GCP per image to one GCP every second image. Based on these contrasting findings, one may conclude that further research is needed. Thereby, the prediction and description of GCP visibility is essential.

One way to describe GCP visibility in images is the recover frequency of GCPs for *k* visible GCPs ($\nu _{{\text {GCP}}}(k)$), determined by the ground field of view (*G*), the exposure stations of the camera ($I_{{\mathrm {Pos}}}$) and the position of the GCPs (${\text {GCP}}_{{\mathrm {Pos}}}$),

26 $$\begin{aligned} \nu _{{\text {GCP}}}(k)&= \frac{1}{I_n} \mathop \sum \limits _{i=1}^{I_n} \left[ \mathop \sum \limits _{j=1}^{{\text {GCP}}_n} \left[ {\text {GCP}}_{j,{\text {Pos}}} \in \left( I_{i,{\text {Pos}}} \pm \frac{G}{2} \right) \right] = k \right] , \nonumber \\ {\text {for}}\, k&= 0, 1, \ldots , N. \end{aligned}$$

For the spatial arrangement of GCPs, we differentiate between two common arrangements: squared and crosswise. The squared arrangement is favorable in situations where the number of rows that a GCP may be placed in is restricted. The crosswise arrangement has its advantage in increasing the recover frequency while at same time reducing the number of needed GCPs.

#### Viewing geometry

In close-distance remote sensing, viewing geometry effects may influence the resulting photos significantly [19, 39, 41–43]. The viewing geometry depends on spacing between flight lines and exposure stations, but also on the design of the examined area, e.g. the number and spacing of plots in an agricultural field experiment. A field experiment with a rectangular design may be specified by the centers of plots ($p_{{\mathrm {Pos}},x}$, $p_{{\mathrm {Pos}},y}$). To control viewing geometry effects and reach a high frequency of pixels in a desired view (e.g. close nadir views) in relation to these plot centers, one may calculate the recover frequency of plot centers for each pixel,

27 $$\begin{aligned}&a \in \left\{ - \frac {S_a}{2}, \ldots , \frac {S_a}{2} \right\} , \; b \in \left\{ -\frac {S_b}{2}, \ldots , \frac {S_b}{2} \right\} \end{aligned}$$

28 $$\begin{aligned}&\nu _{p}(a) = \frac{1}{I_n \cdot p_n} \mathop \sum \limits _{i=1}^{I_n} \mathop \sum \limits _{j=1}^{p_n} f_1 \left( p_{j,{\text {Pos}},x} - \left( I_{i,x,{\text {Pos}}} + a \cdot {\text {GSD}} \right) \mid \sigma _{I_{{\mathrm {Pos}}}} \right) \end{aligned}$$

29 $$\begin{aligned}&\nu _{p}(b) = \frac{1}{I_n \cdot p_n} \mathop \sum \limits _{i=1}^{I_n} \mathop \sum \limits _{j=1}^{p_n} f_2 \left( p_{j,{\text {Pos}},y} - \left( I_{i,y,{\text {Pos}}} + b \cdot {\text {GSD}} \right) \mid \sigma _{I_{{\mathrm {Pos}}}} \right) \end{aligned}$$

30 $$\begin{aligned}&\nu _p(a, b) = \nu _p(a) \cdot \nu _p(b) , \end{aligned}$$

where $\sigma _{I_{{\mathrm {Pos}}}}$ represents the standard deviation of the positioning precision, $p_n$ the number of plots, and $f_1$ and $f_2$ the positioning precision distribution of the UAS. For the positioning of flight lines, one may assume a standard normal distribution ($f_1(x) = X \sim {\mathcal {N}}\left( 0,\sigma ^{2}\right)$). For the positioning of exposure stations along flight lines, a uniform distribution may be more appropriate ($f_2(x) = X \sim \mathcal {U}(-\frac {\sigma }{2},\frac {\sigma }{2})$), based on the assumption that an initial positioning error at the start of a line is propagated from exposure to exposure.

The zenith angle for pixels in relation to observed plot centers may be calculated as

31 $$\theta (a, b) = \frac {\pi }{2} - \tan ^{-1}\left( \frac{f}{S_{\delta } \cdot \sqrt{\left( a - \frac {S_a}{2}\right) ^2 + \left( b - \frac {S_b}{2}\right) ^2 }} \right).$$

Equations 30 and 31 will therefore yield a distribution of zenith angle frequencies $\nu _\theta (\theta )$,

32 $$\begin{aligned}&\nu _\theta (\theta _i) = \mathop \sum \limits _{a,b} \nu _p(a,b) \; {\text { for all }} \; \theta (a,b) \in [\theta _i,\theta _i+1 ), \theta _i \in \mathbb {N}_0. \end{aligned}$$

#### Way-point flight mapping missions

A common way to map an area with an UAS is to perform way-point flights while sequentially trigger the camera. This technique allows to capture photos in-flight, which significantly saves flight time in comparison to flights where each exposure station is represented by a way-point where the UAS hovers to capture a photo [40, 44]. Therefore, in this we focus on in-flight capture techniques.

The location-independent parameters of a mapping flight where defined in section ‘Mapping areas’. To perform a way-point flight at a specific location, three additional location parameters are required: ${P}_{{\mathrm {Start}}}$ represents the point where the UAS takes off. For most UAS, this point additionally defines the reference altitude. ${P}_{{\mathrm {Edge1}}}$ represents one edge of the mapping area, while ${P}_{{\mathrm {Dir}}}$ defines the flight direction: ${P}_{{\mathrm {Edge1}}}$ and ${P}_{{\mathrm {Dir}}}$ form a primary flight direction baseline which all flight lines are aligned with.

Mapping an uneven ground may significantly influence the resulting GSD: UASs usually hold a flight elevation that is relative to the elevation of the starting point (${P}_{{\mathrm {Start}}}$), which results in varying flight heights in relation to the ground. As a consequence, recent way-point flight tools allow to perform follow-terrain-flights. While a true follow-terrain-flight would require a device to measure the distance to the ground [45], tools like Litchi (VC Technology Ltd, London, England) simulate the same behavior by adapting individual heights of way-points using a digital elevation model. Prerequisite for such follow-terrain flights is that the number of way-points along the flight route is sufficient to capture terrain differences. This requirement is in contradiction to the artificially introduced limitation of number of way-points ($P_{n,\max }$) for some UASs, e.g. the maximum of 100 way-points for UASs from DJI (SZ DJI Technology Co. Ltd., Shenzhen, China).

## Implementation

### Architecture

*PhenoFly Planning Tool* is programmed in R [46] using R Shiny [47] as web application framework. The tool depends on the public available packages ggplot2 [48], gridExtra [49], NMOF [50, 51], RJSONIO [52], rlist [53], rgdal [54], readr [55], zoo [56], data.table [57] and raster [58]. *PhenoFly Planning Tool* therefore runs on every platform that supports R—it was tested to run locally on both windows and linux systems. Using an R Shiny server to provide the app to users completely overcomes operating system borders and offers full functionality to any client running a web-browser that supports JavaScript.

### Graphical user interface

**Fig. 4:**
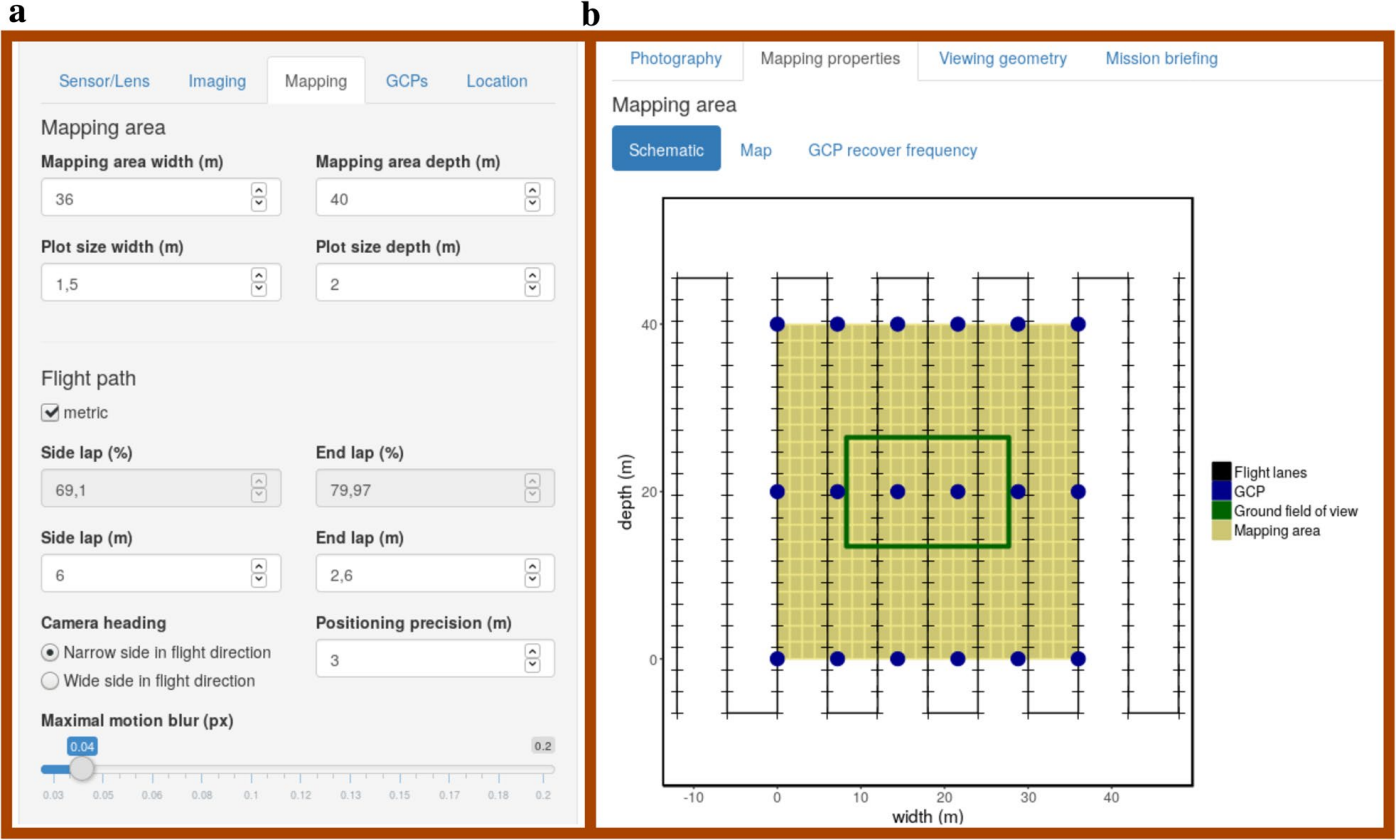
*PhenoFly Planning Tool* graphical user interface (GUI) layout with **a** side panel for inputs and **b** main panel for outputs

The graphical user interface (GUI) is divided in a side panel to allow the user to specify input values (Fig. 4a) and a main panel to show calculated output values, graphical illustrations and summaries (Fig. 4b). The input values are grouped according to Tables 1 and 2 in tabs named *Sensor/Lens*, *Imaging*, *Mapping*, *GCPs* and *Location*. The content of the output tabs *Photography*, *Mapping Properties*, *Viewing Geometry* and *Mission Briefing* are described in detail below.

#### Photography

**Fig. 5:**
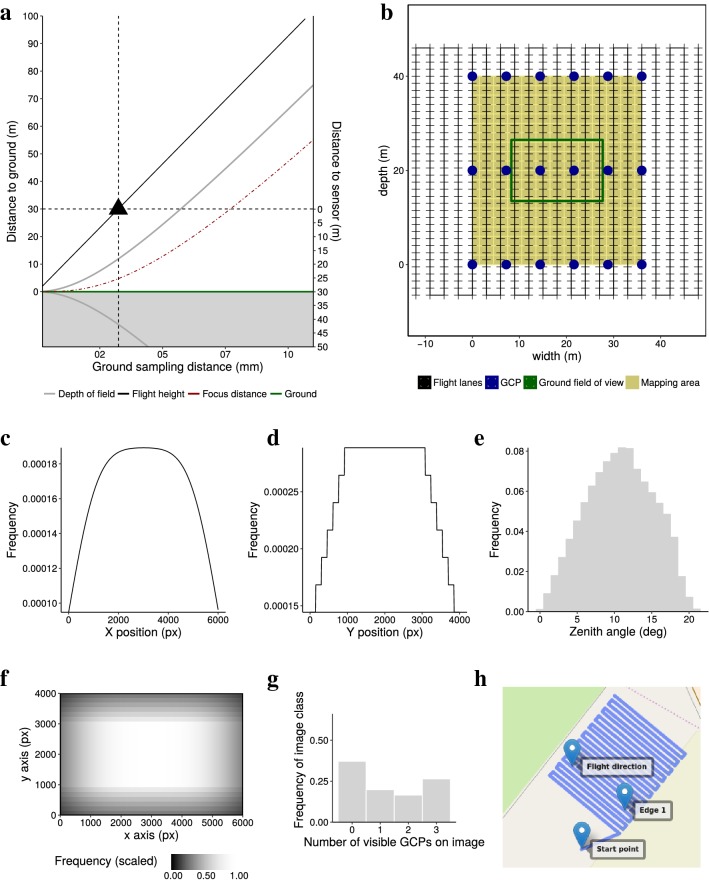
The graphical user interface (GUI) output components of the *PhenoFly Planning Tool* for the following tabs: photography: **a** influence of flight height on ground sampling distance, focus distance and depth of field. **b** Mapping properties: schematic mapping area. **c**–**f** Viewing geometry: frequency of plot center recover for sensor width (x-axis, **c**), sensor height (y-axis, **d**) and individual sensor pixels (**f**), and resulting zenith angle frequency for plot centers (**e**). **g** GCP: ground control point recover frequency distribution. **h** Way-point flight: Geospatial implementation of mapping flight

The photography output tab presents a graph illustrating the relationship between flight height and resulting GSD (Eqs. 6, 7) (Fig. 5a). The aperture f-number is fixed according to the input tab *Sensor/Lens*, allowing to display additional information about optimal focus distance (Eq. 10) and resulting depth of field in relation to the ground (Eq. 8). Additional information about lens intrinsic parameters—angle of view (Eq. 5) and hyperfocal distance (Eq. 3)—are noted aside the graph.

#### Mapping properties

The mapping properties output tab displays information on the implementation of a mapping mission with a way-point flight (Eqs. 14–20). On the first page, a schematic graph shows the mapping area, the flight path and the GCP arrangement (Fig. 5b). Aside the graph, mapping parameters for the implementation—photo recording speed and photo trigger interval (Eq. 23), flight speed (Eq. 25), minimum flight duration (Eq. 22), and number of photos (Eq. 21)—are noted.

On the second page, the flight path is presented in a geospatial context showing the to-implement way-point flight with start point, edge of mapping area and flight direction (Fig. 5h). *PhenoFly Planning Tool* partially supports follow-terrain functionality: the tool intersects flight lines with additional way-points while as same time respecting a limit of $P_{n,\max }$ way-points.

The third page allows the users to optimize their GCP arrangement to reach the required recover frequency: the GCPs are automatically placed on the mapping area to best suite the given GCP recover frequency. The placement algorithm thereby favors equal distances between GCPs and penalizes high number of required GCPs. After the automatic placement, the user can further customize the arrangement by changing the arrangement pattern or manually increase and decrease the number of GCPs in both directions. A graph and table show the recover frequency of GCPs in images based on the chosen settings (Eq. 26) (Fig. 5g).

#### Viewing geometry

The viewing geometry output tab is divided in two parts: the left part is denoted to a sensor-centered view, while the right part represents a plot-center-centered view. The sensor-centered view presents a graph showing the frequency of plot centers imaged on certain pixel positions (Eq. 30) (Fig. 5c and d for the two sensor axis, Fig. 5f for individual pixels). The plot-center-centered view presents the frequency of plot views having a certain zenith angle (Eq. 32) (Fig. 5e).

#### Mission briefing

The mission briefing tab summarizes all fight-relevant information. The tab additionally provides the user with the possibility to download a report containing all graphs and a JSON file containing all entered parameters for documentation purpose. To continue the flight campaign workflow in an third-party-tool, two export possibilities are provided: first the possibility to export the mapping area as KML file for sub-sequential import in tools that allow to map an area based on a shape (e.g. DJI GS Pro) and second a possibility to export the way-points as way-point CSV file for import it in a tool that allows to import way-point flights (e.g. Litchi, VC Technology Ltd, London, England).

## Results

### Comparison with other recent mission planning tools

To contrast the presented software *PhenoFly Planning Tool* with comparable tools, we performed a systematic evaluation of current available mission planning and way-point flight tools. We focused on classical ground station software packages that are able to generate mapping flights. A selection of ten tools is listed in [59], based on which we performed an intense internet research to validate and update this list.

Thereby, we evaluated the functionality using two techniques: (1) For software that was available for an affordable fee (open source, free of charge or small fee of less than $25 USD), we did the evaluation based on the software itself. This category included the following tools:

- Aerobotics flight planner tower v4.0.1 (https://github.com/DroidPlanner/Tower)
- Altizure v4.1.0 (https://www.altizure.com/mobile)
- DJI GS Pro v1.8.1 (https://www.dji.com/ground-station-pro)
- Drone Harmony Mission Planner v0.8.1 (http://droneharmony.com)
- DroneDeploy v2.69.0 (https://www.dronedeploy.com/app.html)
- Litchi v4.4.0 (https://flylitchi.com)
- Map Pilot for DJI v2.8.0 (https://www.mapsmadeeasy.com)
- MikroKopter Tool v2.20 (http://wiki.mikrokopter.de/en/MikroKopterTool)
- Mission Planner v1.3.55 (http://ardupilot.org/planner/index.html)
- Pix4Dcapture v3.0.0 (https://pix4d.com/product/pix4dcapture)
- QGroundControl v3.3 (http://qgroundcontrol.com)

For software that was priced higher than $25 USD, but the documentation was publicly available, we evaluated the functionality based on the documentation. This category included:

- Autopilot for DJI Drones v4.4 (http://autoflight.hangar.com)
- UgCS v2.13 (https://www.ugcs.com/)

We did not consider software that was priced higher than $25 USD if the documentation was not publicly available. Software that was available only as integral part of an UAS/software package without a freely accessible documentation was handled in the same category. This category included (non-exclusive):

- DroneLogbook (https://www.dronelogbook.com)
- eMotion (https://www.sensefly.com/software/emotion)
- MAVinci (http://www.mavinci.de/de/mavinci-system/mission-control-software)
- mdCockpit App v2018.07.h2 (https://www.microdrones.com/de/mdaircraft/software/mdcockpit-app/)
- Skyward (https://skyward.io)
- Unifly Pro (https://www.unifly.aero/products/unifly-pro)

The evaluation was divided in three parts: (1) Type of tool, (2) calculations and (3) features. In the type of tool classification part (1) we categorized the tools by their mean purpose. If a tool matched a certain category, it was marked with the rating “x”.

In the calculation evaluation part (2) we assessed the tools regarding their informativeness. More precisely, we analyzed whether a tool considered a specific parameter or not, and if this was the case, whether the tool handled the parameter as user input or provided the user with a calculated output value. If a parameter was handled as input, it was rated with an “I”, if it was handled as calculated output with an “O”. Implementations where the user could choose whether to handle a parameter as input or calculated output were rated as “I/O”.

In the features evaluation part (3), we classified the tools regarding useful features that may increase their utility in comparison with others. If a feature was implemented, we rated the category as “x”. The rating “no information” indicated that we where—based on the documentation—not able to determine whether a feature was implemented or not. For the supported UAS and operation system categories, we listed the specific systems using number and letter keys.

The results of the evaluation are summarized in the overview Table 3. In the following, we discuss this results according to the categories type of tool, calculations and features.

**Table 3:**
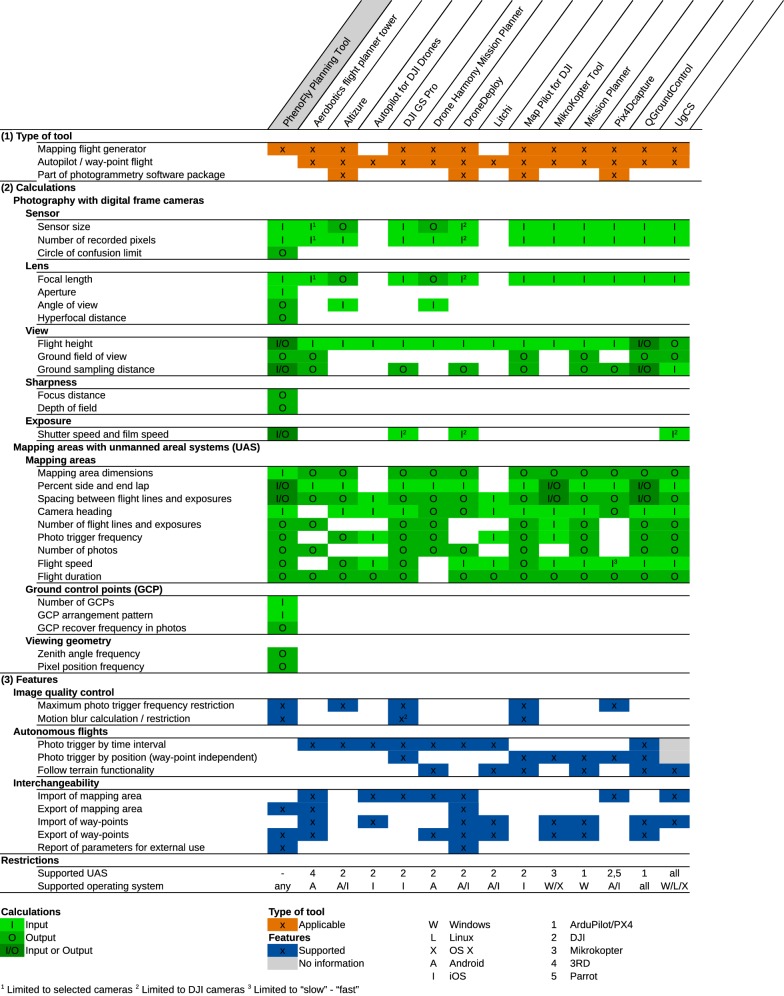
Evaluation results, divided in the categories (1) type of tool, (2) calculations and (3) features

#### Type of tool

All but the *PhenoFly Panning Tool* are so called “online-tools” in the meaning of being able to control an UAS using autopilot functionality [59], while four of them are additionally part of a photogrammetry software package (Table 3). *PhenoFly Planning Tool* on the other hand is the only tool that does not provide such autopilot functionality and may therefore be called an “offline-tool”. Two tools (Autopilot for DJI Drones and Litchi) are pure autopilot apps, while all other tools additionally support mapping flight generation.

The finding that *PhenoFly Panning Tool* is the only offline-tool version of all evaluated software tools supports our hypothesis that current developments are strongly bound to specific vendors and operating systems. Despite that offline-tools were presented by different authors over time [e.g. 60, 61], the availability of executable binaries or even source code for those tools is very restricted. This limitation also applies to the evaluated third-party tools: in particular tools that support DJI systems tend to be closed-source software, while tools that support the Mikrokopter and ArduPilot universe are almost exclusively open source tools. *PhenoFly Panning Tool* therefore adds to the urgent need for vendor-neutral, well-documented evaluation and flight preparation tools.

#### Calculations

*PhenoFly Planning Tool* includes critical photogrammetric properties for flight planning and supports input parameters of all frame-camera and thin-lens combinations, which contrasts other tools: only three out of 14 evaluated tools (DJI GS Pro, UgCS and DroneDeploy) do consider shutter speed settings and therefore may control image quality by setting a maximum motion blur value (Table 3). Unfortunately, for all of the three mentioned tools, image quality control only works in combination with dedicated specific cameras. As motion blur is a factor influencing image quality severely (see results of Experiment 2 in section ‘Ground sampling distance and motion blur’), this lack of control is a major drawback of the evaluated tools.

Regarding mapping parameters, all tools are very comparable to *PhenoFly Planning Tool*, with the exception of Autopilot for DJI Drones and Litchi, which are by definition pure autopilot tools and therefore do not support mapping flights *per se*. Two tools stand out with extended side and end lap calculation support based on percentage values as well as exact spacing values (MikroKopter Tool and QGroundControl). It may be noted that certain tools do not show values in the GUI that were obviously calculated in the back-end: Aerobotics flight planner tower, Pix4DCapture and DroneDeploy for example do not display a photo trigger interval but most probably use the value to trigger the UAS camera in autopilot mode. An adjustment of flight mission parameters based on restrictions of the imaging system is therefore unfeasible in these tools.

GCPs and viewing geometry are only considered by *PhenoFly Planning Tool*. As mentioned in section ‘Mapping areas with unmanned areal systems’, GCP recover frequency has a major influence on the georeferencing precision of resulting photogrammetric products, while viewing geometry effects may bias information extraction from mapping campaigns.

#### Features

The scope of provided features differs largely among tools. The image quality control functionality of *PhenoFly Planning Tool*, DJI GS Pro and Map Pilot for DJI are comparable, with the advantage of the two later tools to have the possibility to directly implement a flight using the internal autopilot. Autonomous flight features are largely supported among tools. Most tools are able to perform photo triggering by time or position, although many tools only support either triggering by time or distance. Follow terrain functionality becomes more and more common: almost half of all evaluated tools support terrain corrected flights based on digital elevation models.

Regarding interchangeability, some tools stand out with extensive import and export possibilities (e.g. DroneDeploy and Aerobotics flight planner tower), while others have no open interface at all (e.g. Map Pilot for DJI and Altizure). The support for certain operating systems and UAS vendors varies widely: some tools support several operating systems (e.g. QGroundControl and UgCS), but most tools are restricted to one specific operating system for desktop computers (e.g. Mission Planner) or tablets (e.g. DJI GS Pro). All presented tools are specific for one or two UAS vendors, with the exception of the universal tool UgCS.

### Application demonstration

To validate the usability of the described concepts and the *PhenoFly Planning Tool* in real-world conditions we performed different field trials using a Matrice 600 Pro (SZ DJI Technology Co. Ltd., Shenzhen, China) (approximated maximum flight time: 15 min.) as UAS and a Sony $\alpha 9$ (ILCE-9, Sony Corporation, Tokio, Japan) (sensor size: 35.6 $\times$ 23.8 mm, number of recorder pixels: 6000 $\times$ 4000, maximum photo trigger frequency: 2 s$^{-1}$, maximum tolerable ISO: 4000) combined with a Sonnar$^{\textregistered }$ T* FE 55 mm F1,8 ZA lens (Sony Corporation, Tokio, Japan) as RGB imaging device. The camera was connected to the UAS using a Ronin-MX (SZ DJI Technology Co. Ltd., Shenzhen, China) gimbal to prevent off-nadir views and rotation blur effects caused by abrupt movements of the UAS.

In Experiment 1, we performed flights at varying flight heights in sunny and windless weather conditions to visualize the effect on GSD and visibility of details in images (Table 4). The flight speed was kept at a constant rate of 1 m s$^{-1}$. Shutter speed was set to 1/2500 s, ISO to 350 and aperture to f/6.7.

**Table 4:** Parameters for the GSD experiment (Experiment 1): effects of flight height on ground sampling distance (GSD) and motion blur in pixel % for a constant flight speed of 1 m s$^{-1}$ and a shutter speed of 1/2500 s

Flight height	19 m	28 m	46 m	93 m
GSD (mm)	2	3	5	10
Motion blur (%)	20	13	8	4

In Experiment 2, we performed flights at constant height with varying flight speeds in overcast but windless weather conditions to visualize motion blur effects in low-light conditions (Table 5). Flight height was set to 46 m, shutter speed to 1/500 s, ISO to 320 and aperture to f/8.0. For both Experiment 1 and 2, we imaged UV coated GCP prints with the size of 0.2 $\times$ 0.2 m, a DIN A4 sized IT 8.7 color checker panel (http://www.targets.coloraid.de, Wolf Faust, Frankfurt, Germany) with 10 $\times$ 10 mm sized color boxes, and an experimental wheat plot (*Triticum aestivum* L.) in BBCH stage 31/32 [62].

In Experiment 3, we planned and performed a complete mapping flight on a wheat experiment, including GCP placement. The base requirements were to map an area of 40 $\times$ 35 m with a resulting GSD of 3 mm, a maximal tolerable motion blur of 5%, and one or more visible GCP in more than 75% of all images. Targeted image overlaps were proportional to plot sizes (3 $\times$ 1.5 m overlap versus 1 $\times$ 1.5 m plot sizes). The wide sensor side ($S_x$) should point in flight direction. The mapped area was not flat but curved with a height difference > 4 m between the highest and lowest point. In addition, the area was bordering an obstacle with a height of 25 m at one corner, i.e. the winch-tower of the field phenotying platform of ETH Zürich (FIP) [63]

**Table 5:** Parameters for the motion blur experiment (Experiment 2): effects of flight speed on motion blur in pixel % for a flight height of 46 m and shutter speed of 1/500 s

Flight speed	4 m/s	8 m/s	10 m/s	15 m/s
Motion blur (%)	160	320	400	600

*PhenoFly Planning Tool* proposed a mapping flight at 28 m height with a flight speed of 1.8 m s$^{-1}$ if setting shutter speed to 1/16,000 s, ISO to 2500 and aperture to f/5.6. The calculated focus distance was 25.7 m. Estimated flight duration was 8 minutes, estimated number of photos to take was 741. A crosswise GCP arrangement with 6 $\times$ 3 GCP led to the targeted percentage of 75% photos with one or more GCP per photo. The mapping flight was performed using DJI GS Pro (SZ DJI Technology Co. Ltd., Shenzhen, China) as autopilot.

Captured photos were processed using structure-from-motion (SfM) and image projection techniques to determine exposure stations and viewing geometries of images. Details of the method can be found in [19]. In brief, Agisoft PhotoScan Professional 1.4.2 (Agisoft LLC, St. Petersburg, Russia) was used to process individual photos, delivering a digital elevation model (DEM), but also exposure station, GSD, flight height and image overlap estimations. Thereafter, images were projected to the DEM using ray-tracing techniques, and masked using individual plot masks for each image. This process resulted in viewing geometry information (zenith angle) for all visible plots and GCPs on processed images.

Flights were performed on April 20 (Experiment 1, GSD), Mai 2 (Experiment 2, motion blur) and March 25 (Experiment 3, mapping flight), 2018.

#### Ground sampling distance and motion blur

**Fig. 6:**
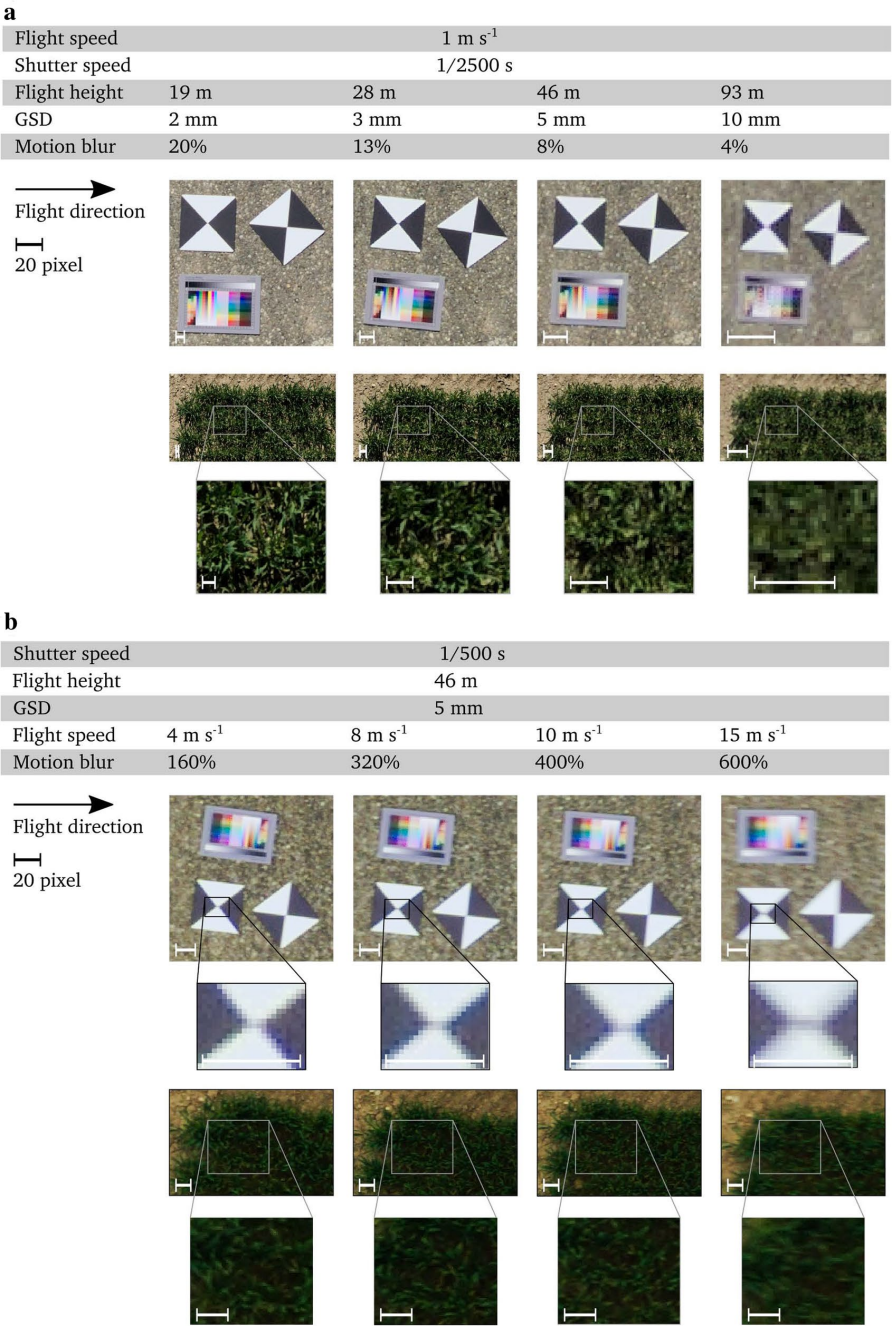
Ground sampling distance (GSD) and motion blur dependency on flight speed, shutter speed and flight height

Experiment 1 (various flight heights in sunny conditions) resulted in photos with differing GSDs, but also level of visible details (Fig. 6a): for a GSD of 2 mm, individual color fields of the color checker panel image were clearly visible. Individual leafs in the wheat vegetation image were distinguished and clearly separated from the background. For a GSD of 3 mm, borders between color fields in the color checker panel image softened, and the separation between individual plant leafs in the wheat vegetation image vanished. For a GSD of 5 mm, the increase of mixed pixels hampered a clear separation in plant and background segments, and for a GSD of 10 mm, the majority of pixels were mixed pixels and a separation in plants and soil therefore not possible. The drastic drop in level of visible details with increasing GSD stresses the importance of an adequate equipment and the determination of a suitable flight height in flight preparation.

Experiment 2 (low-light conditions with varying flight speed but constant flight height) resulted in photos with comparable GSD, but differing visible motion blur effects (Fig. 6b). Due to lower lighting conditions, the contrast in photos was remarkably lower than in Experiment 1. Strong blurring effects became visible in both GCP and vegetation photos starting at $\delta >400\%$. Nevertheless, at $\delta >160\%$, deformation effects—visible by the stretched white space of the GCPs—were already detectable. In vegetation photos, blurring effects in photos with a motion blur ${<}\,$ 400% were only to a small extend visible. This finding emphasize the need to determine and control motion blur in flight preparation, as quality control mechanism on resulting images may be difficult to implement.

**Fig. 7:**
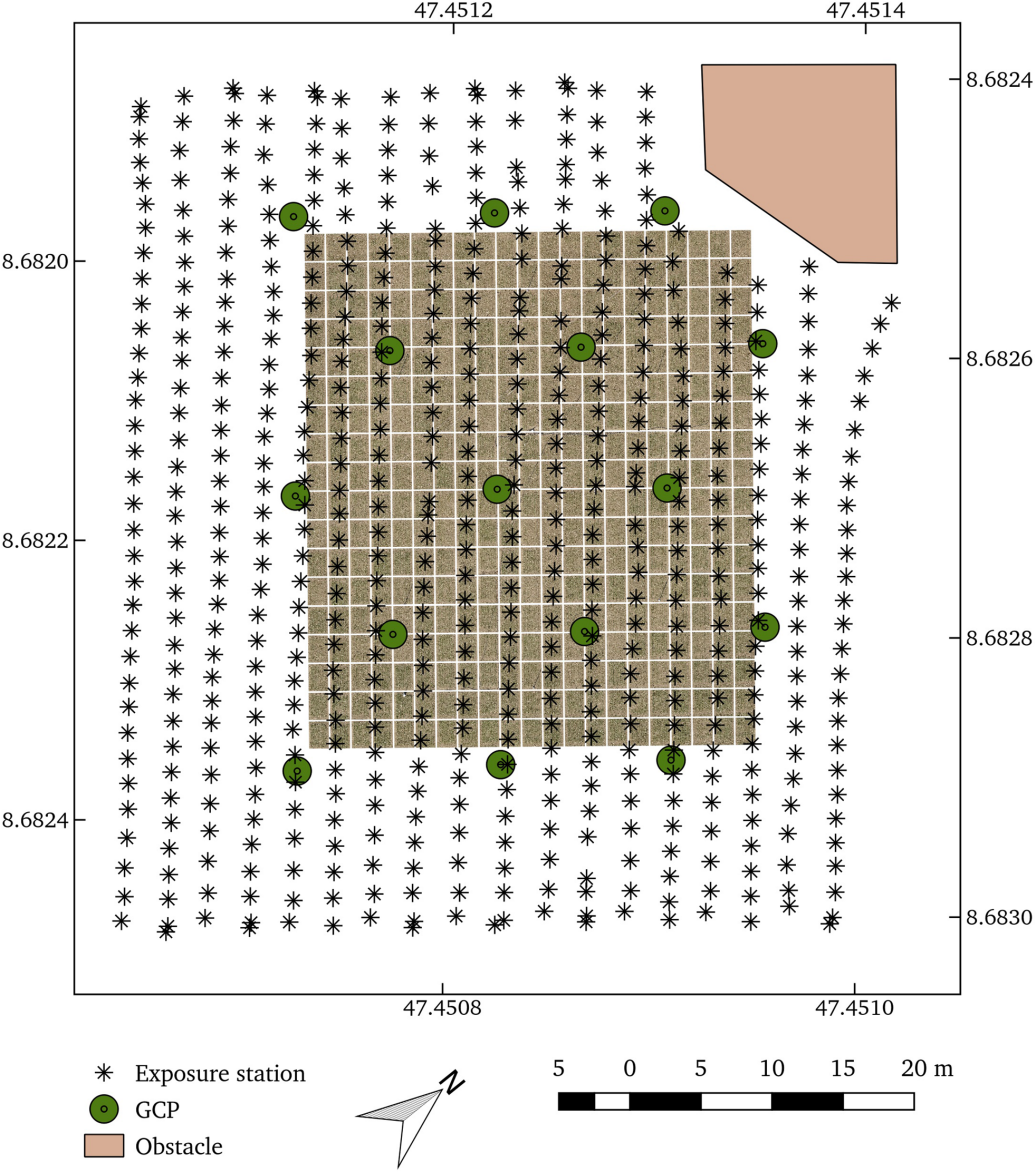
Realized path of the mapping flight for Experiment 3. Indicated are exposure stations (stars), ground control points (GCPs) (green circles), winch-tower in the right corner of the mapping area (brown polygon) and examined field plots (brown squares)

#### Mapping flight

Experiment 3 (complete mapping flight using DJI GS Pro as autopilot) resulted in the flight path visualized in Fig. 7. Flight lines deviated regarding orientation and straightness. The photo trigger interval appeared to be more constant. As DJ GS Pro does not allow to import way-point files directly, we were forced to use the KML shape import functionality and the built-in algorithm of DJ GS Pro to generate way-point flights. As a consequence, the implemented flight deviated regarding the proposed parameters of *PhenoFly Planning Tool*, and the flight lines did not precisely match the corners of the mapping area. Therefore, the number of photos and flight lines were reduced (Table 6). Additionally, the calculated flight duration was slightly higher, most probably because DJI GS Pro adds additional time buffers in turning points. Despite the deviation of two minutes, the estimated flight duration was a valuable element in flight preparation and resulted in the flight being successfully completed while considering the battery capacity range of the UAS. Enhancing DJ GS Pro with *PhenoFly Planning Tool* provided us with the advantage of staying with the flight control software of the manufacturer, which is most likely the safest to control the UAS.

**Table 6:** Predicted and realized flight and mapping parameters and ground control point (GCP) recover frequency for Experiment 3

Parameter	Predicted	Realized
Number of photos	741	572
Flight duration (min)	8	10
Flight height (m)	28	28.4
Number of flight lines	19	18
GSD	3.00 mm	3.02 mm
Overlap	92 × 75%	> 89% (overall)
GCP recover frequency		
0 per image (%)	21	26
1 per image (%)	52	45
2 per image (%)	27	29

Processing reports from Agisoft Photoscan indicated that the targeted GSD of 3.00 mm was reached, despite the uneven ground. The spacing between flight lines and exposure stations led to an overall overlap of > 89%, which is in accordance to the predefined requirements. The GCP recover frequency deviated slightly from the targeted frequency: 26% photos had no visible GCP, which is 5% higher than predicted. Further investigations showed that unreliable automatic detection of GCPs in edges of the sensor were main cause for this deviation. If the GCP in image edges were manually added, the percentage of photos without a visible GCP decreased to 23%, which corresponds to the targeted maximum value of 25%. This results confirmed that taking into account GCP distribution as early as in the experimental setup phase may ensure sufficient GCP recover frequencies in final remote sensing missions.

**Fig. 8:**
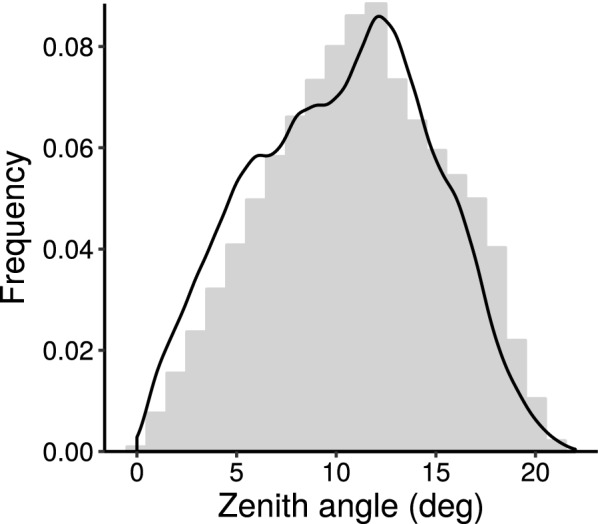
Frequency distribution of plot-center based zenith angles for the prediction (gray surface) and realization (black line) in Experiment 3

In addition to GCP recover frequencies, we examined the plot center recover frequencies. In Experiment 3, viewing geometries (zenith angles) for plot centers were comparable with the predicted frequency distribution of *PhenoFly Planning Tool* (Fig. 8): the shape of the frequency distribution in the implemented flight correspond to the shape of the predicted distribution, while the values were slightly shifted to more close-nadir views by 2$^{\circ }$–$3^{\circ }$. This distribution confirms that close-nadir views rarely happen if performing mapping flights. Nevertheless, predicting viewing geometries in flight preparation may allowed to estimate the level of uncertainty introduced, or to plan flights that will later become processed with multi-view techniques [19, 64].

## Conclusion

Qualitative characteristics of photos taken by UAS have a major influence on the usability of the data (e.g. feature detection and high-resolution segmentation). Image quality is influenced by exposure (as function of shutter speed, aperture and ISO), ground sampling distance (as function of flight height and sensor resolution), and many other factors such as motion blur (as function of flight speed, shutter speed and GSD), viewing geometry (as function of angle of view), spacing between exposure stations, flight lines and examined objects.

Current flight planning and mapping tools for UASs strongly focus on vendor-specific solutions and have no dedicated focus on photographic properties. In this publication, we outlined the most important aspects to be regarded for high-quality data collection, and provided with *PhenoFly Planning Tool* an interactive learning environment to access these concepts. The software thereby bridges the gap between UAS based mission planning and involvement of photographic properties and provides the community with a tool-set to improve tasks such as:

1. Performing a pre-purchase evaluation of UASs including (external) camera systems
2. Assess the requirements and feasibility of a planned mission, thereby reducing uncertainty regarding photographic products
3. Optimize flight parameters to meet the requirements for dedicated mapping missions
4. Set-up experiments with optimized GCP distribution, plot size and viewing geometry
5. Prepare flights and import the calculated parameters into a sophisticated autopilot system (for example Litchi as lightweight way-point flight tool, or UgCS as full-featured autopilot tool)
6. State hardware and flight parameters and derived quality parameters as metadata to published studies and datasets

With the publication of the free software *PhenoFly Planning Tool* (https://shiny.usys.ethz.ch/PhenoFlyPlanningTool), we hope to provide a tool that will increase the efficiency and success of UAS-based remote sensing flights, but also complement this publication for learning purpose.
